# The cholinergic anti-inflammatory pathway ameliorates acute viral myocarditis in mice by regulating CD4^+^ T cell differentiation

**DOI:** 10.1080/21505594.2018.1482179

**Published:** 2018-09-03

**Authors:** Zhou De-Pu, Ge Li-Sha, Chen Guang-Yi, Gu Xiaohong, Xing Chao, Zheng Cheng, Zhang Wen-Wu, Li Jia, Lin Jia-Feng, Chu Maoping, Li Yue-Chun

**Affiliations:** a Department of Cardiology, The Second Affiliated Hospital and Yuying Children’s Hospital of Wenzhou Medical University, Wenzhou, China; b Department of Pediatric Emergency, The Second Affiliated Hospital and Yuying Children’s Hospital of Wenzhou Medical University, Wenzhou, China; c Children’s Heart Center and Department of Pediatrics, The Second Affiliated Hospital and Yuying Children’s Hospital of Wenzhou Medical University, Wenzhou, China; d Department of Clinical Laboratory, The Second Affiliated Hospital and Yuying Children’s Hospital of Wenzhou Medical University, Wenzhou, China; e Department of Intensive Care Unit, The Second Affiliated Hospital and Yuying Children’s Hospital of Wenzhou Medical University, Wenzhou, China

**Keywords:** Cholinergic anti-inflammatory pathway, viral myocarditis, inflammatory cytokines, CD4^+^ T cells, Th cell subsets

## Abstract

Many studies have found that abnormalities in the proportion and differentiation of CD4^+^ T cells (Th cells) are closely related to the pathogenesis of viral myocarditis (VMC). Our previous research indicates that the cholinergic anti-inflammatory pathway (CAP) attenuates the inflammatory response of VMC and downregulates the expression of cytokines in Th1 and Th17 cells. This suggests that the cholinergic anti-inflammatory pathway likely attenuates the inflammatory response in VMC by altering Th cell differentiation. The aim of this study is to investigate the effect of CAP on CD4^+^ T cell differentiation in VMC mice. CD4^+^ T cells in the spleen of VMC mice were obtained and cultured in the presence of nicotine or methyllycaconitine (MLA). Cells were harvested and analyzed for the percentage of each Th cell subset by flow cytometry and transcription factor release by Western blot. Then, we detected the effect of CAP on the differentiation of Th cells in vivo. Nicotine or MLA was used to activate and block CAP, respectively, in acute virus-induced myocarditis. Nicotine treatment increased the proportion of Th2 and Treg cells, decreased the proportion of Th1 and Th17 cells in the spleen, reduced the level of proinflammatory cytokines, and attenuated the severity of myocardium lesions and cellular infiltration in viral myocarditis. MLA administration had the opposite effect. Our result demonstrated that CAP effectively protects the myocardium from virus infection, which may be attributable to the regulation of Th cell differentiation.

## Introduction

The pathogenesis of viral myocarditis (VMC) can be divided into three stages. In the first stage, viral infection directly injures the cardiomyocytes while inducing the innate immune response of the host to eliminate pathogens. In the second stage, myocardial necrosis from the first stage induces inflammatory cells to attack normal myocardium, augmenting the injury. In the third stage, there is a wide range of immune injury to cardiomyocytes and myocardial fibrosis, resulting in progression to dilated cardiomyopathy [1]. Many studies have indicated that CD4^+^ T cells and their cytokines played a critical role in the last two stages [2]. Recently, many researchers found that CD4^+^ T cell subsets, including Th1, Th2, Th17, and Treg, play an important role in viral myocarditis, and the functions of each subgroup varied in myocarditis. Huber and others discovered that male mice who mostly had a Th1 cell-mediated immune response were more susceptible to the CVB3 virus than female mice who had a Th2-mediated immune response [3]. Rangachari et al. reported that Th17 cells and its cytokine, IL-17, increased the severity of viral myocarditis and autoimmune myocarditis [4]. Treg cells could inhibit the expression of inflammatory cytokines and attenuate the severity of viral myocarditis [5]. Therefore, it is important to regulate Th cell differentiation in viral myocarditis.

Two elements determines the differentiation of naive T cells, the specific transcription factors and cytokines in the local microenvironment. A variety of cytokines are activated in the pathogenesis of viral myocarditis. These cytokines are closely related to Th cell differentiation. Interestingly, these cytokines also have major overlap with the cytokines regulated by the cholinergic anti-inflammatory pathway (CAP). The CAP is a recently proposed immunoregulatory pathway that inhibits the release of inflammatory cytokines by connecting the nervous system to the immune system, ameliorating the inflammatory response of many diseases, such as sepsis, ulcerative colitis, and rheumatoid arthritis. Our previous studies have indicated that the CAP significantly decreases the level of Th17 cell-related IL-17A and IL-6 as well as Th1 cell-associated TNF-α [6–10]. Galitovskiy et al. also found that α7-nicotinic acetylcholine receptor (α7-nAChR) agonist, nicotine, increased the ratio of Treg cells and reduced the ratio of Th17 cells, improving the prognosis of ulcerative colitis [11]. These results suggest that CAP may regulate the differentiation of CD4^+^ T cells in VMC mice.

Although the anti-inflammatory effect of CAP on VMC has been demonstrated in previous studies from our group and others [6–8,12], it has not been reported whether CAP can affect the differentiation of CD4^+^ T cell subsets. However, based on previous studies, we hypothesized that CAP could inhibit the inflammatory response by modulating the differentiation of Th cells subsets in VMC, and reducing myocardium lesions. Therefore, this study will focus on the regulation effect of CAP on Th cell subsets.

## Results

### Effects of nicotine or methyllycaconinitine on the regulation of spleen CD4^+^ T cell differentiation in vitro

**Figure 1.**
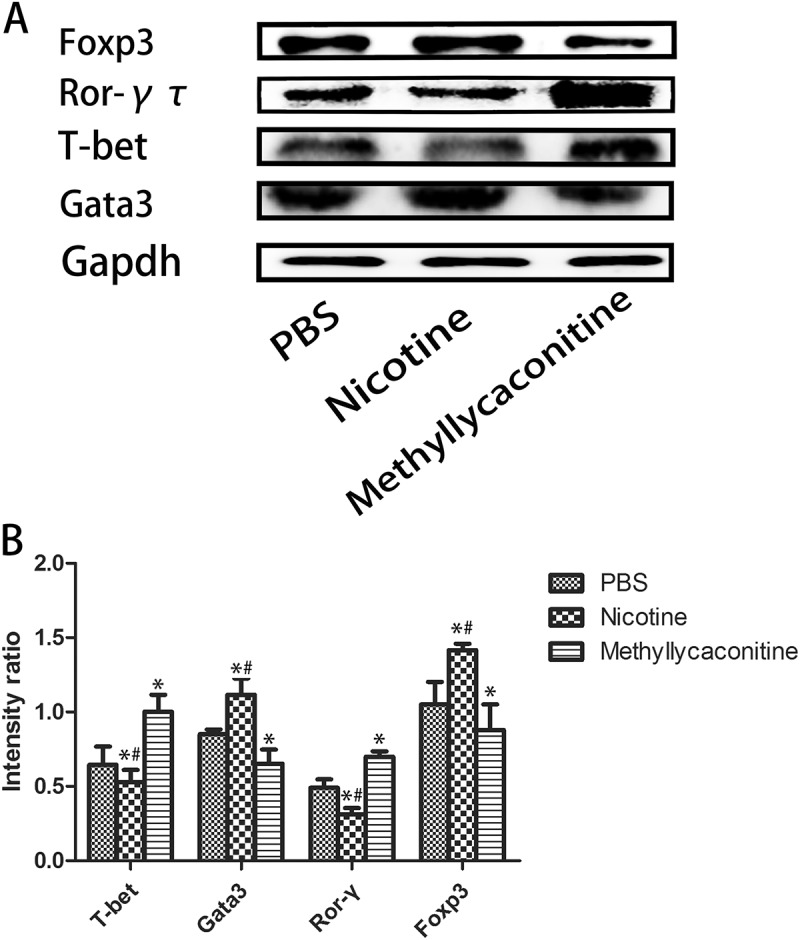
The expression of Th cell-associated specific transcription factors in the spleen cells from CVB3-infected mice (n = 5 in each group). Nicotine upregulated GATA3 and Foxp3 expression, while it downregulated T-bet and ROR-γ expression. A. Representative levels of the specific transcription factors. B. Absolute intensity ratio of the specific transcription factors to Gapdh. *P < 0.05 versus the PBS group and ^#^P < 0.05 versus the methyllycaconitine group.

T-bet, GATA3, ROR-γ and Foxp3 are the specific transcription factors of Th1, Th2, Th17 and Treg cells, respectively; therefore, we analyzed the distribution of Th1, Th2, Th17 and Treg cells in the spleens of control and VMC mice [13]. Nicotine treatment could upregulate GATA3 and Foxp3 expression while downregulating T-bet and ROR-γ expression compared to the PBS group. (P < 0.05) However, the expression levels of GATA3 and Foxp3 were reduced, and the expression levels of T-bet and ROR-γ were elevated in the methyllycaconitine (MLA) group in the separate CD4^+^ T cells. (P < 0.05) (Figure 1)

### Effects of nicotine or methyllycaconinitine treatment on the percentage of CD4^+^ T cells in the spleen in vitro

**Figure 2.**
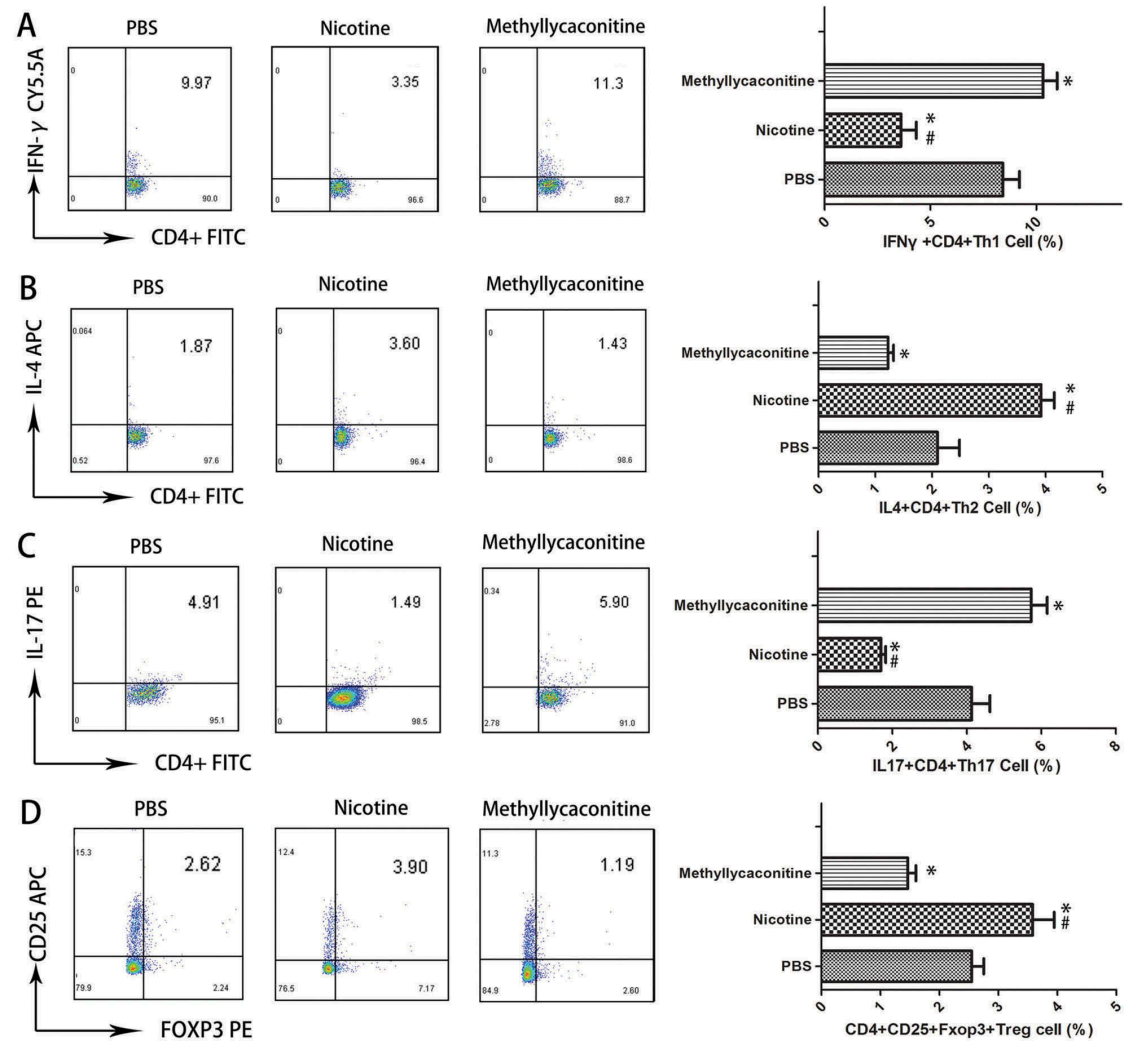
The percentage of Th cell subsets in the spleen from CVB3-infected mice (n = 5 in each group). The percentages of IFN-γ^+^ CD4^+^ Th1 and IL-17^+^ CD4^+^ Th17 cells were increased in the MLA group, but they were decreased in the nicotine group. CD4, IFN-γ, IL-4, IL-17, CD25 and Foxp3 were labeled by FITC, PE-CY5.5, APC, PE, APC, and PE, respectively. A, B, C and D are the representative results for each group and the percentage of double positive cells. *P < 0.05 versus the PBS group and ^#^P < 0.05 versus the methyllycaconitine group.

Then, we used flow cytometry to detect the percentage of the Th subgroup in separated CD4^+^ T cells from CVB3-induced mice. The percentages of IFN-γ^+^ CD4^+^ Th1 and IL-17^+^ CD4^+^ Th17 cells were increased in the MLA group, but they were decreased in the nicotine group. (P < 0.05) However, the percentages of IL-4^+^ CD4^+^ Th2 and CD4^+^ CD25^+^ Foxp3^+^ Treg cells were increased in the nicotine group and decreased in the MLA group. (P < 0.05) (Figure 2)

### Survival rate

**Figure 3.**
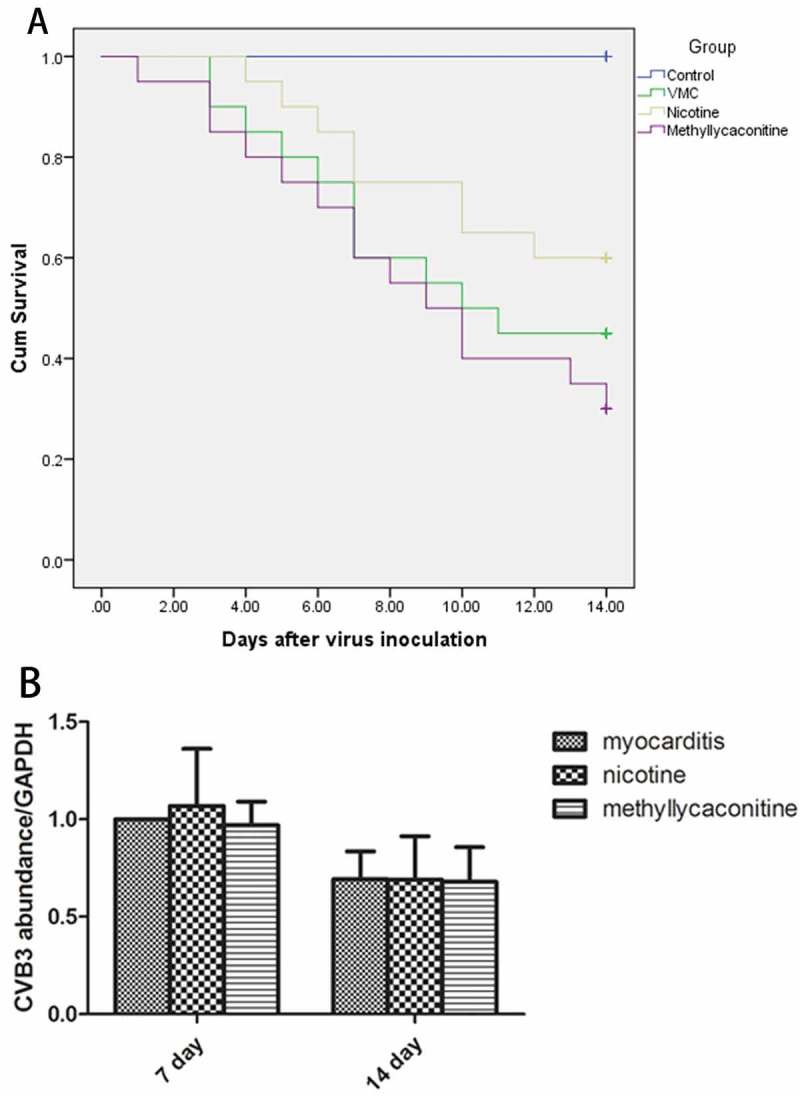
Survival rate (n = 40 in each group) and Viral replication in the myocardium (n = 5 in each group). A. The survival curve of each group. B. Viral replication in the myocardium of each group.

BALB/C mice were injected with 10^5^ TCID_50_ of CVB3 virus by intraperitoneal injection to generate a viral myocarditis model. The mice injected with CVB3 had weakness, weight loss, back arching and irritability. There was no death in the control group after 14 days; 45% (18 of 40) mice survived in the VMC group. The survival rate of the nicotine group was 60% (24 of 40), and it was 30% (12 of 40) for the MLA group. The survival rate was significantly decreased in the VMC and MLA groups compared with the control group (each P < 0.05 versus the control group), but no significant change in the survival rate was seen in the nicotine group compared with the control group (P = 0.062 versus the control group). Compared with the VMC and MLA groups, the survival rate was slightly increased by nicotine, but this effect did not reach statistical significance (P = 0.282 versus the VMC group; P = 0.054 versus the MLA group). (Figure 3)

### Myocardial histopathology

**Figure 4.**
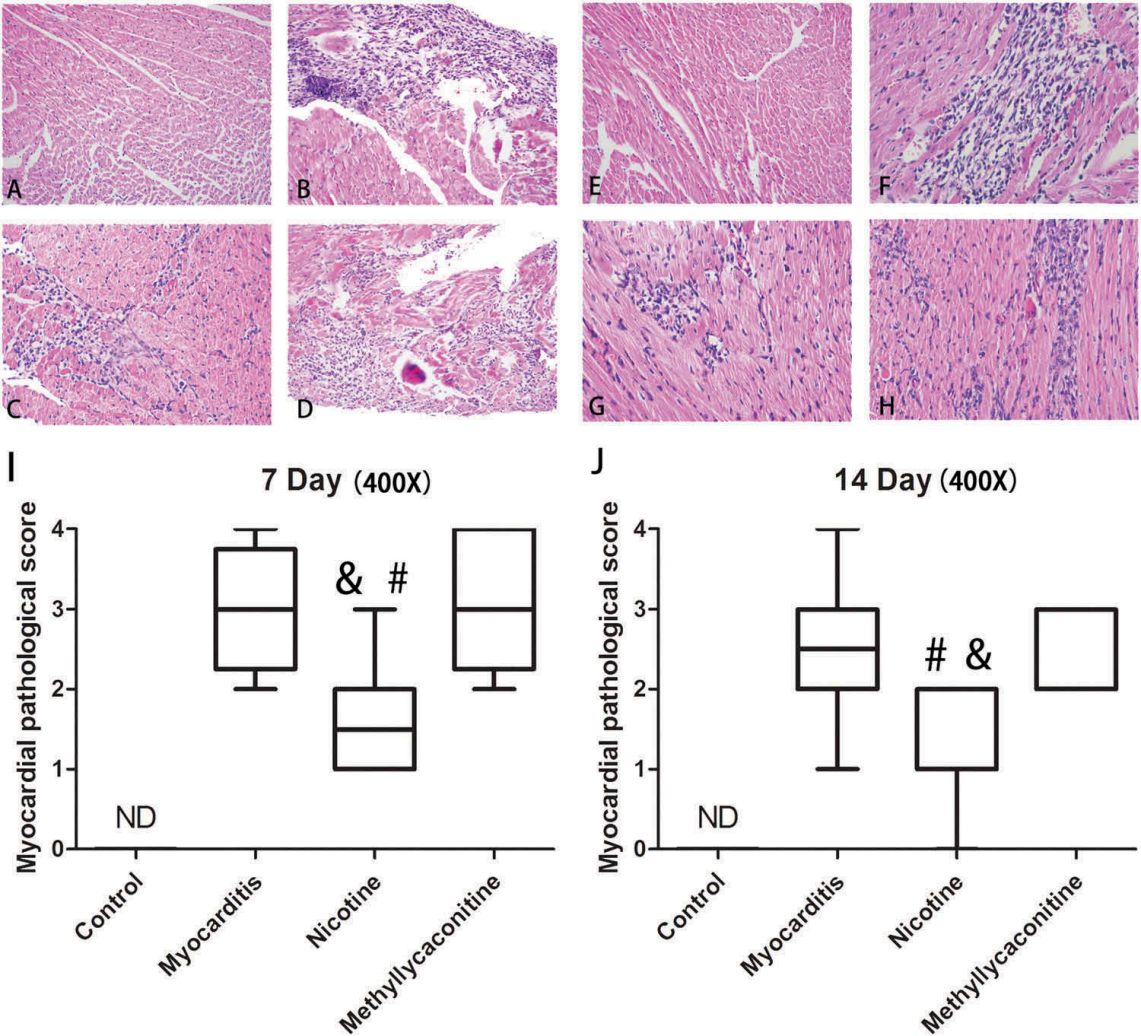
Myocardial histopathology changes for each group (n = 5 in each group). A and E show the lack of histopathology changes for the control group on days 7 and 14; B and F show representative histopathology changes for the myocarditis group on days 7 and 14. Several small foci of cellular infiltrations are shown; C and G show representative histopathological changes for the nicotine group on days 7 and 14. The extent of cellular infiltration was less severe in the nicotine group than in the methyllycaconitine and myocarditis groups; small and limited foci of cellular infiltrations were obtained. D and H show representative histopathological changes for the methyllycaconitine group on days 7 and 14. Several large foci of cellular infiltrations are obtained. I and H are the Box-plots of the histopathology scores for each group on days 7 and 14. The score of the nicotine group was significantly lower than those of the myocarditis and methyllycaconitine groups. ^&^P < 0.05 versus the myocarditis group and ^#^P < 0.05 versus the methyllycaconitine group.

The mice that survived were sacrificed on days 7 and 14. The myocardium of the VMC group showed a severe injury with cellular infiltration and necrosis. Nicotine alleviated the severity of cellular infiltration and necrosis compared with the VMC group. (P < 0.05) (Figure 4). There was no significant difference in histological score between the VMC group and the MLA group.

### Viral replication in the myocardium

We detected the CVB3-RNA abundance in the myocardium of the VMC, nicotine and MLA groups by fluorescent quantitative PCR-analysis. There were no significant differences between the treatment and VMC groups on days 7 and 14. (Figure 3)

### The levels of the pro-inflammatory cytokines in the heart on days 7 and 14

**Figure 5.**
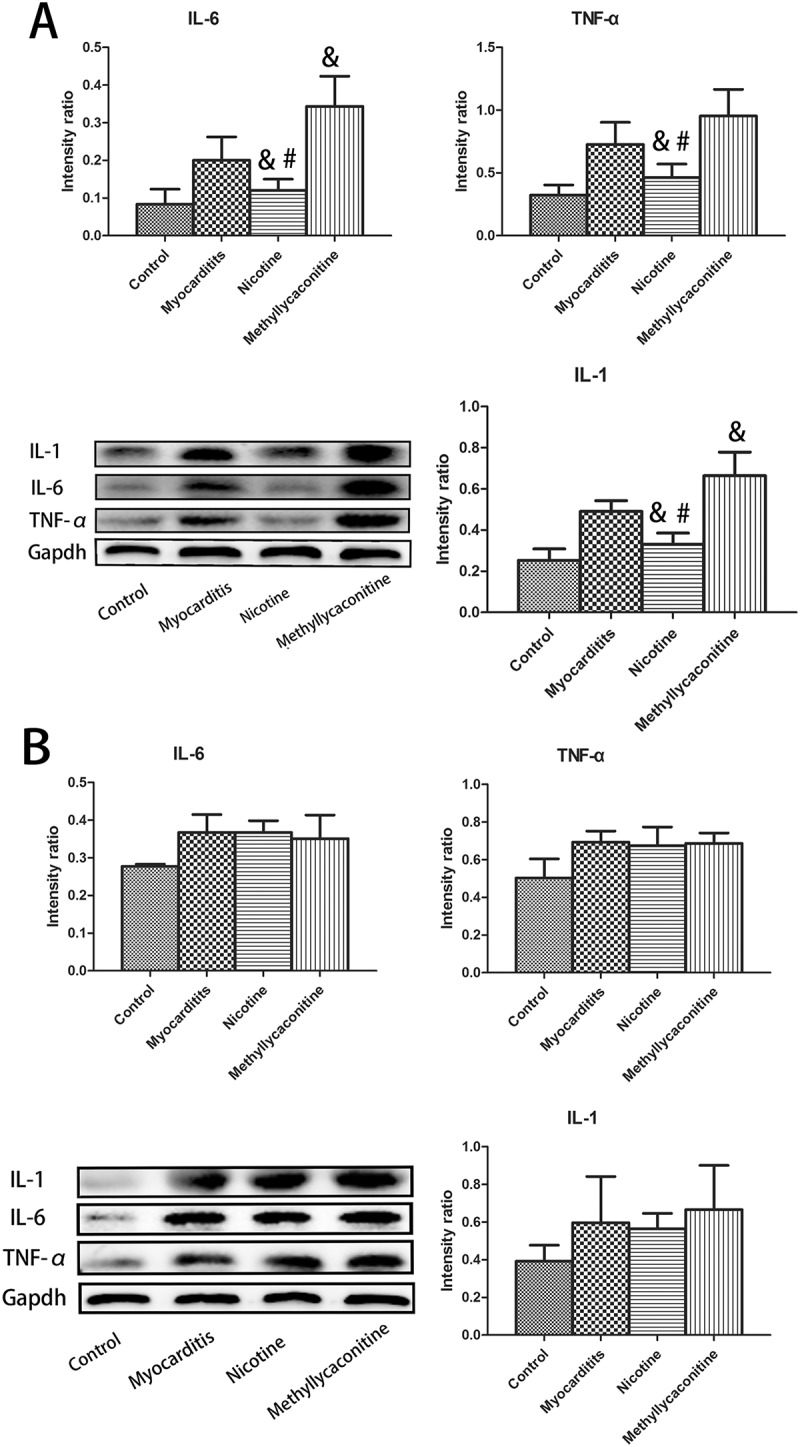
Expression of pro-inflammatory cytokines in the heart (n = 5 in each group). A. Nicotine treatment significantly deceased the expression of IL-1, IL-6 and TNF-α compared with the myocarditis group on day 7. B. On day 14, no significant differences were found in the level of pro-inflammatory cytokines among the nicotine group, myocarditis group and methyllycaconitine group. ^&^P < 0.05 versus the myocarditis group and ^#^P < 0.05 versus the methyllycaconitine group.

Then, we examined the expression of IL-1, IL-6 and TNF-α for each group on days 7 and 14. On day 7, the levels of IL-1, IL-6 and TNF-α were significantly lower in the nicotine group than in the VMC group. (P < 0.05) However, the expression of those pro-inflammatory cytokines was elevated in the MLA group compared to the VMC group. (P < 0.05) On day 14, no significant differences were found in the level of pro-inflammatory cytokines among the Nicotine group, VMC group and MLA group. (P > 0.05) (Figure 5)

### The expression of specific transcription factors and cytokines in a Th cell subset in the heart on days 7 and 14

**Figure 6.**
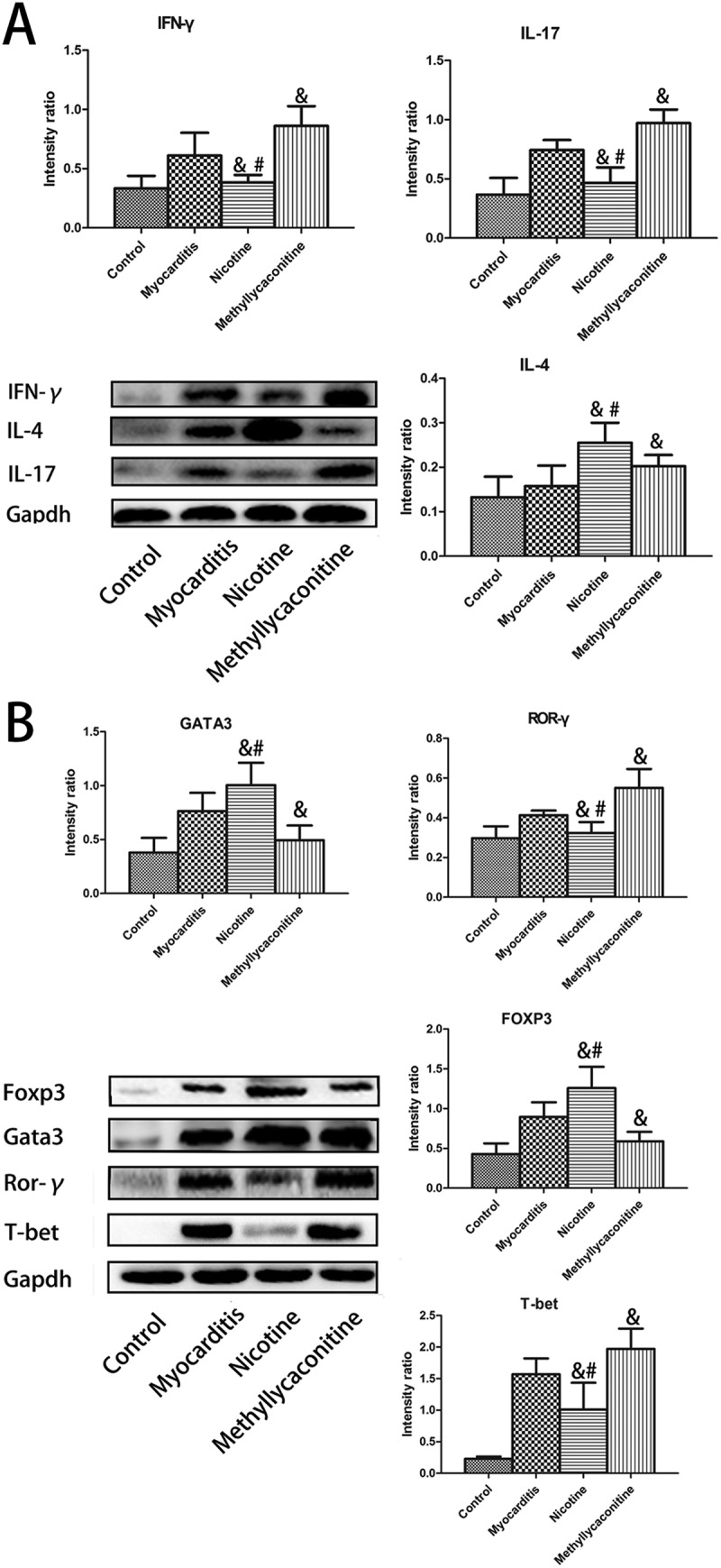
Expression of specific transcription factors and cytokines for Th cell subsets in the heart on day 7 (n = 5 in each group). The levels of IFN-γ, IL-17, T-bet and ROR-γ were lower in the nicotine group than in the myocarditis group. Otherwise, the levels of GATA3, Foxp3 and IL-4 were higher in the nicotine group than in the myocarditis group. A is the absolute intensity ratio of the cytokines for the Th cell subset. B is the absolute intensity ratio of the specific transcription factors. ^&^P < 0.05 versus the myocarditis group and ^#^P < 0.05 versus the methyllycaconitine group.

**Figure 7.**
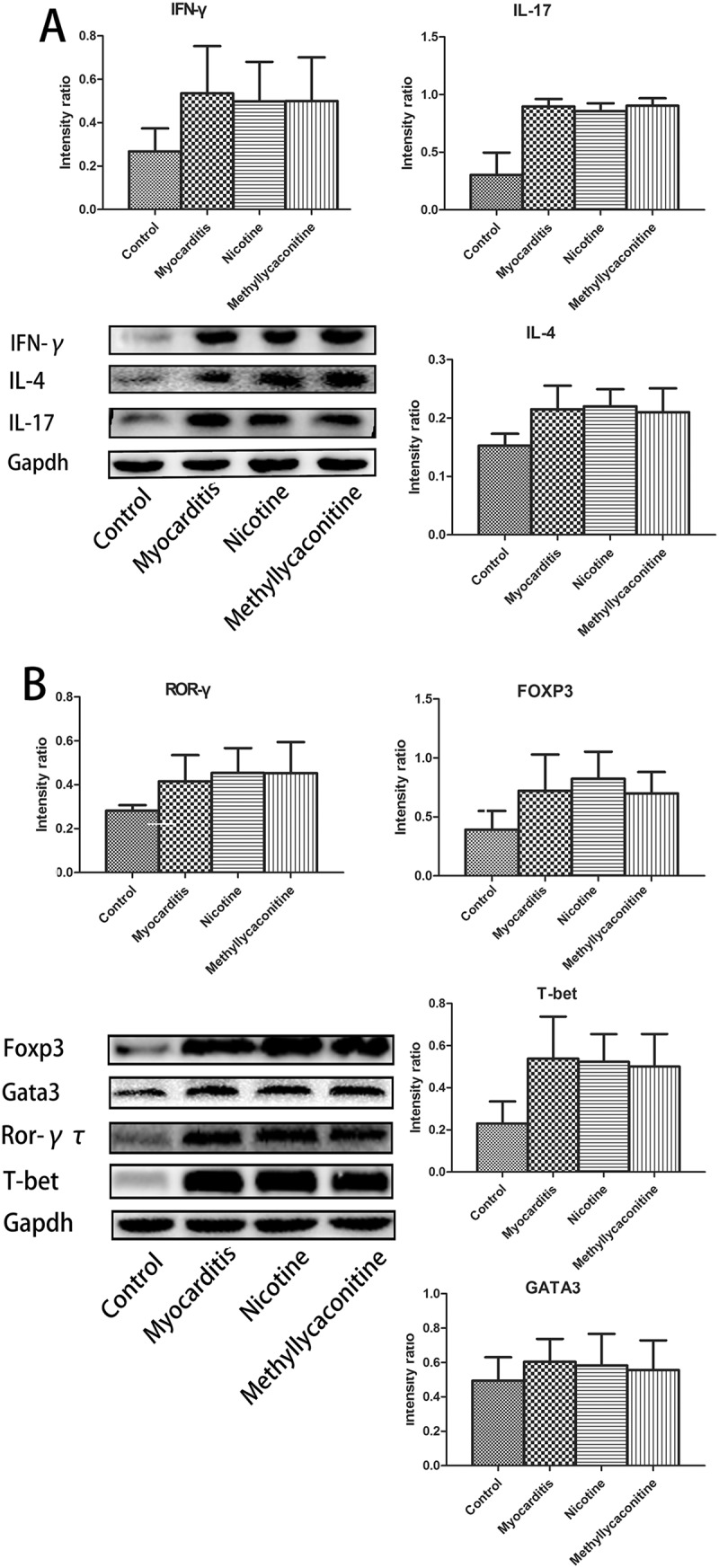
Expression of specific transcription factors and cytokines of the Th cell subset in the heart on day 14 (n = 5 in each group). There was no significant difference between the treatment and myocarditis groups. A is the absolute intensity ratio of cytokines for the Th cell subset. B is the absolute intensity ratio of the specific transcription factors. ^&^P < 0.05 versus the myocarditis group and ^#^P < 0.05 versus the methyllycaconitine group.

IFN-γ, IL-4, IL-17 are specific cytokines for Th1, Th2 and Th17 cells, respectively; therefore, we could observe the percentage variations of Th1, Th2, Th17, and Treg cells with changes in the levels of specific transcription factors and cytokines of the heart for each group. On day 7, the levels of IFN-γ, IL-17, T-bet and ROR-γ were lower in the nicotine group than in the VMC group. Otherwise, the levels of GATA3, Foxp3 and IL-4 were higher in the nicotine group than in the VMC group. (P < 0.05) However, the MLA group had the opposite result. Compared with the VMC group, the expression levels of IFN-γ, IL-17, T-bet and ROR-γ were elevated, and the expression levels of GATA3, Foxp3 and IL-4 were decreased. (P < 0.05) (Figure 6) On day 14, there was no significant difference between the treatment and VMC groups. (Figure 7)

### The percentage of th subsets in the spleen in vivo

**Figure 8.**
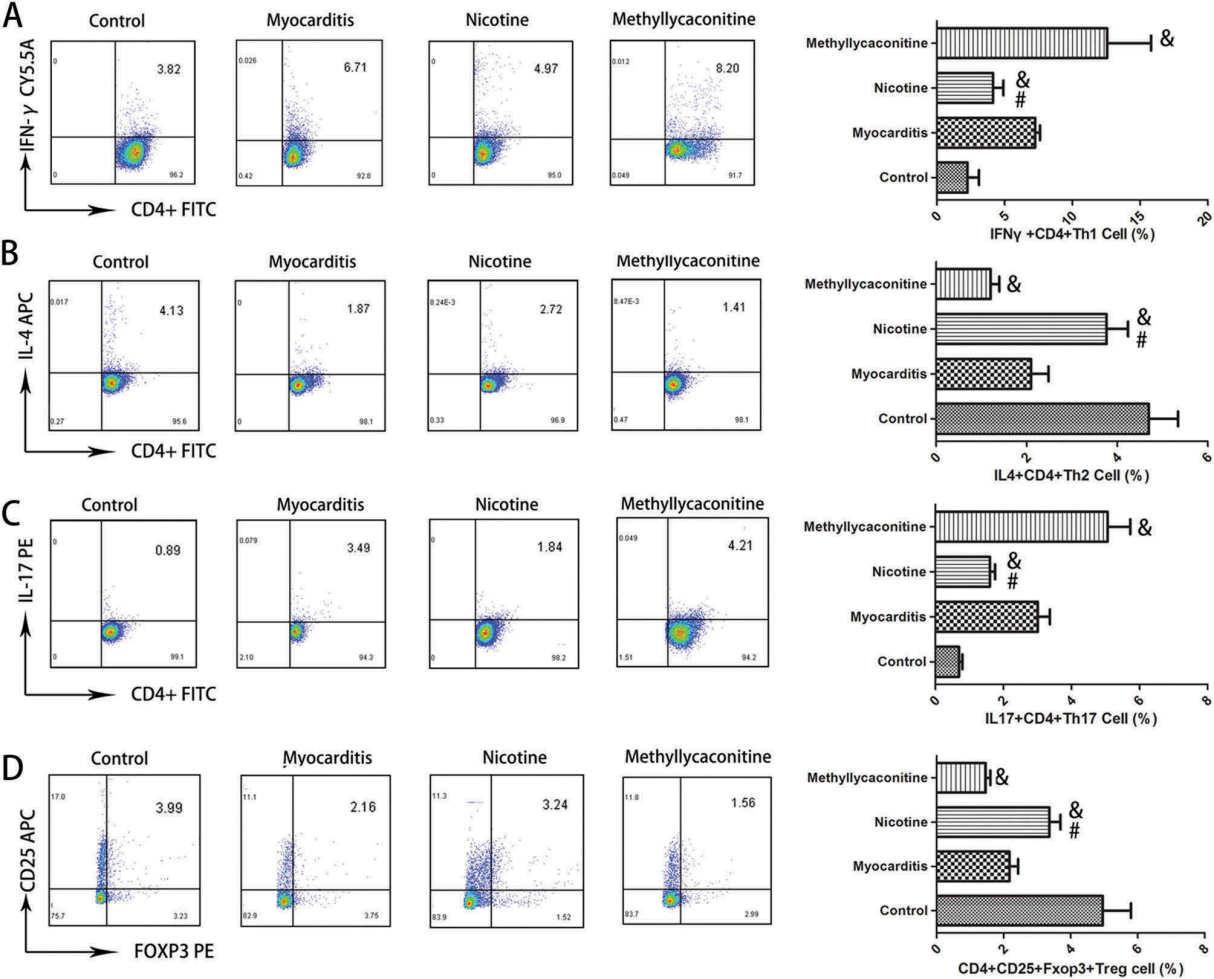
The percentages of Th subsets in the spleen (n = 5 in each group). On day 7, the percentages of Th1 and Th17 cells were deceased, while the percentages of Th2 and Treg cells were increased in the nicotine group. ^&^P < 0.05 versus the myocarditis group and ^#^P < 0.05 versus the methyllycaconitine group.

The percentage of Th subsets in the spleens of control and VMC mice was analyzed. Compared with the VMC group, the percentages of Th1 and Th17 cells decreased, and the percentages of Th2 and Treg cells increased in the nicotine group on day 7.(P < 0.05) Compared with the VMC group, the percentages of Th1 and Th17 cells were elevated and the percentages of Th2 and Treg were decreased in the MLA group. (P < 0.05) (Figure 8)

## Discussion

The results of this study indicated the regulatory effect of the CAP on the differentiation of CD4^+^ T cells in viral myocarditis. First, stimulation of spleen cells from VMC mice with nicotine in vitro altered the percentage of Th cell subsets by increasing the percentage of Th2 and Treg cells and decreasing the percentage of Th1 and Th17 cells. Second, nicotine treatment in vivo altered the percentages of Th cell subsets in the spleen in a similar manner. The change in the spleen resulted in variations in the specific transcription factors and cytokines expression in the myocardium. The expression levels of Th2-specific transcription factor GATA3 and Treg cells-specific transcription factor Foxp3 were elevated, and the expression levels of Th1-specific transcription factor T-bet and Th17-specific transcription factor ROR-γ were reduced in the myocardium. Finally, nicotine treatment reduced inflammatory cytokines (IL-1β, IL-6, and TNF-α) in the myocardium, ameliorated the necrosis of cardiomyocytes and cellular infiltration in mice with viral myocarditis.

The CAP, which is composed of the vagus nerve, acetylcholine secreted by the vagus nerve terminal and receptor α7 acetylcholine receptors, is important. Infection and ischemia release cytokines, activate signals, and transfer factors into the nucleus tractus solitarius via the vagus nerve and through the cranial nerve network, activating the release of acetylcholine on the vagus nerve. Acetylcholine activates α7 nicotinic acetylcholine receptors on macrophages, lymphocytes and other inflammatory cells, regulating the synthesis and release of inflammatory cytokines and attenuating the systemic inflammatory response [14]. Compared with traditional humoral immunity, the CAP is extremely sensitive and rapid, with accurate localization of the inflammatory response to the infected tissue. The CAP had an anti-inflammatory effect at the early phase of the inflammatory response [12]. However, the protective effect disappeared after splenectomy [15]. The spleen was the intermediate link for the CAP in which CD4^+^ T cells and their subsets played an important role in the anti-inflammatory function. Many studies have shown that the CAP could regulate the differentiation of CD4^+^ T cells by inhibiting the release of inflammatory cytokines, improving the prognosis of inflammatory diseases. Wu et al. found that the CAP reduced the percentage of Th17 cells, increased the percentage of Th2 cells and attenuated the inflammatory response of arthritis in mice [16]. Nizri et al. also demonstrated that nicotine treatment inhibited the response of Th1 and Th17 cells and attenuated neuroinflammation in autoimmunity encephalomyelitis [17]. Nicotine treatment could ameliorate inflammatory bowel disease severity through increasing the proportion of Treg cells and decreasing the proportion of Th17 cells [11]. In this research, we proposed that the CAP could regulate the differentiation of CD4^+^ T cells in viral myocarditis. The results revealed that nicotine could downregulate the expression of T-bet and ROR-γ as well as upregulate the expression of GATA3 and Foxp3 in CD4^+^ T cells. The results of flow cytometry were also consistent with those of WB. Nicotine treatment decreased the percentages of Th1 and Th17 cells and increased the percentages of Th2 and Treg cells in the spleen. The proportions of Th1 and Th17 cells were increased and the proportions of Th2 and Treg cells were decreased in the MLA group. These results demonstrated that the CAP could regulate the differentiation of spleen CD4^+^ T cell subsets, decrease the percentages of Th1 and Th17 cells and increase the proportions of Th2 and Treg cells.

In the first two stages of the process of viral myocarditis, there are many mechanisms involved in injury to the myocardium, including the following four features: 1. direct injury with virus infection and replication; 2. virus-specific immunity; 3. adaptive immunity induced by viral replication; and 4. autoimmunity [18]. CD4^+^ T cells and their subgroups mediate adaptive immunity, participating in the development and progression of viral myocarditis. For Th17 cells, substantial evidence had demonstrated that they are involved in the pathogenesis of inflammatory diseases. It had been found that anti-IL-6 treatment could reduce the proportion of Th17 cells, reducing the inflammatory reaction of autoimmune myocarditis [19]. In viral myocarditis, Yang et al. found that administration of recombinant IL-23 reduced the percentage of Th17 cells and inflammation in mice [20]. Similarly, IL-27 inhibited the differentiation of Th17 cells, ameliorated the symptoms and improved of the survival rate of viral myocarditis in mice [21]. However, the effect of Th2 and Treg cells is unclear, although many studies have shown that both Th2 and Treg cells could attenuate the inflammatory response and play a protective role in inflammatory diseases. It had been discovered that female mice, which predominantly have Th2 cell immune responses, are less likely to develop viral myocarditis [22]. This is reported that Treg cells were also involved in the susceptibility differences. The monocytic myeloid-derived suppressor cells upregulated the percentages of Treg and CD4^+^ IL-10^+^ T cells to reduce the inflammatory response in viral myocarditis [23]. Th2 cell-associated cytokine IL-33 could alleviate the severity of viral myocarditis in TLR3 knock-out mice, increase the percentage of Th2 cells, and prevent the progression from acute myocarditis to chronic myocarditis [24]. Papageorgiou et al. found that type-2 thrombospondin could activate Treg cells, reduce myocardium lesions and improve cardiac dysfunction in CVB3-induced myocarditis [25]. These studies indicated that Th2 and Treg cells play a protective role in viral myocarditis, improving the disease prognosis. However, unlike the Th cell subsets described above, the role of Th1 in viral myocarditis is not fully understood. Jian et al. reported that IFN-α, a Th1 cell-related cytokine, could induce differentiation of splenic T cells to Th1 cells in viral myocarditis mice, which could reduce virus replication and protect the mice [26]. Similarly, IFN-β, aTh1 cell-related inflammatory cytokine, could prevent viral replication and improve the prognosis of viral myocarditis [27]. However, Yue et al. found that mutations in the IP-10 gene could suppress the level of IFN-γ, reducing myocardial lesions and inflammatory infiltration [28]. In addition, they reported that mutations of MCP-1 reduced the mortality, serum markers, and histopathological changes of viral myocarditis mice [29]. There was a divergence of the effect of Th1 cells. IFN-α/β could prevent viral replication, but IFN-γ would increase the inflammatory response. Further research is needed to determine the role Th1 cells play in inflammatory diseases. Based on the above results and theoretical basis, after clarifying the regulation effect of the CAP on the differentiation of splenic CD4^+^ T cells, we further found that nicotine had the effect of the percentage alteration of CD4^+^ T cells subsets on viral myocarditis. In the acute and subacute phases of viral myocarditis, high levels of viral replication led to cardiomyocytes necrosis, activated the acquired immune system of the host, and resulted in infiltration of the myocardial interstitium. The results of WB showed that nicotine treatment could change the expression of Th cell-specific cytokines and transcription factors in the myocardium of mice with viral myocarditis. The levels of Th1 cell-associated T-bet and IFN-γ as well as Th17 cell-associated ROR-γ and IL-17 expression were upregulated. Th2 cell-related GATA3 and IL-4 and Treg cell-related Foxp3 expression levels were downregulated. Thus, we suggested that the CAP could alter the proportion of CD4 + T-lymphocyte subsets in the spleens of mice, and possibly alter the percentage of T cell subgroups in the myocardium interstitium. This change in the proportion of Th cells ultimately reduced the systemic inflammatory response, alleviated myocardial injury, and protected the mice from viral myocarditis.

Interestingly, there were no significant differences in those groups in terms of the abundance of CVB3 RNA in the myocardium. This might be related to inhibition of Th1 cell differentiation. Previous studies indicated that although Th1 cells inhibited viral replication, they increase the systemic inflammatory response. Our study revealed that activation of the CAP decreased the proportion of Th1 cells and IFN-γ, reducing the anti-virus effect and systemic inflammatory response, which resulted in a net a protective effect. In addition, inflammatory cytokines and Th cell-associated transcription factors were not significantly different at 14 days, which might be related to the progression of viral myocarditis. The virus is nearly eliminated in the animal model of viral myocarditis at 14 days; by that time, it is progressing into the chronic phase [30]. Although our previous studies and this experiment demonstrated that the cholinergic anti-inflammatory pathway could have an anti-inflammatory effect in the early phase of inflammation, evaluation of the effect of the CAP in the chronic phase requires further study.

The analyses in vivo at day 7 and day 14 were performed on mice that survived after CVB3 infection in the study. Although the analyzed mice in each group were selected randomly, the study might have the potential of survivor bias, due to excluding mice that die prior to these experimental endpoints from the analysis.

### Conclusion

In this study, we preliminarily found that the cholinergic anti-inflammatory pathway could regulate CD4^+^ T cell differentiation, increase the percentages of Th2 and Treg cells, and decrease the percentages of Th1 and Th17 cells in the spleen. Those effects possible changed the levels of specific transcription factors of CD4^+^ T cell subsets in the myocardium, reduced myocardial lesions and inflammatory infiltration, attenuated the expression of pro-inflammatory mediators in the acute and subacute phases of the viral myocarditis model. This research provides a new understanding of the development and progression of viral myocarditis as well as provides a potential new target for myocarditis treatment.

## Materials and methods

### Mice

Male, specific pathogen-free, 4-week-old BALB/c mice were obtained from the Laboratory Animal Center of Shanghai (Shanghai, China) and were kept in a pathogen-free facility in the experimental animal center of the Wenzhou Medical University. All animals received humane care according to the Guide for the Care and Use of Laboratory Animals published by the US National Institutes of Health (NIH Publication, 8th Edition, 2011). The study protocol was approved by the Wenzhou Medical University Committee on Ethics in the Care and Use of Laboratory Animals.

### Virus

CVB3 (Nancy strain) was maintained in Hep2 cells. A 50% tissue culture infectious dose (TCID50) assay was used to determine the viral titer. BALB/c mice were intraperitoneally inoculated with 0.2 mL of normal saline containing 10^5^ TCID50 of the virus to generate a viral myocarditis model. The control group as intraperitoneally injected with the same dose of normal saline.

### In vitro CD4^+^ T cell culture

CD4^+^ T cells were purified by positive selection using MACS (Miltenyi Biotec, # 130–049-201) from the spleen of 7-day infected VMC mice. The day of cells separation was defined as day 0. All cells were cultured in six-well plates in the presence of nicotine (Sigma-Aldrich Co, #N3876) or methyllycaconitine (Sigma-Aldrich Co, M168). They were randomly divided into three groups, the phosphate buffer solution (PBS) group, nicotine group (100 μmol/L), and MLA group (100 μmol/L). All drugs were administered once daily for 5 consecutive days, starting on day 1. Cells were harvested and subjected to flow cytometry and Western blot analyses.

### In vivo mouse experiment

The day of virus inoculation was defined as day 0. Nicotine (1.2 mg/kg per day) and methyllycaconitine (2.4 mg/kg per day) were intraperitoneally given to VMC mice for consecutive 14 days, starting 24 h after viral inoculation. Meanwhile, the control and VMC groups were intraperitoneally given the same dose of normal saline solution. Eight surviving mice from each group were sacrificed to extract the heart and spleen on days 7 and 14. The spleens were used for flow cytometry analysis and the hearts were divided into three parts. One part was used for Western blot analyses, another for studying myocardial histopathological and the last for q-PCR analysis.

### Survival analysis

We randomly chose 40 mice from each group for a 14-day survival analysis.

### Histopathological analysis and myocarditis scoring

The hearts collected from the CVB3 infected mice on days 7 and 14 were fixed in 10% formalin overnight. After fixation, the heart tissues were placed in a series of alcohol for dehydration and then embedded in paraffin. Sections (5 μm thick) of hearts tissues were cut and stained with hematoxylin and eosin (H&E). Pathological scores were blindly graded by two independent observers based on the following semi-quantitative scale: 0 = no lesion; 1 = lesion involving 25% of the myocardium; 2 = lesions involving 25 to 50% of the myocardium; 3 = lesions involving 50 to 75% of the myocardium; and 4 = lesions involving 75 to 100% of the myocardium, as previously described [31]. The scores for every section were averaged.

### Flow cytometry

Purified CD4^+^ T cells were counted and suspended in RPMI 1640 containing 10% fetal bovine serum at a density of 1 × 10^6^ cell/ml. To analyze the percentages of Th1, Th2 and Th17 cells, the cells were stimulated for 6 h with leukocyte activation cocktail (BD Pharmingen, #550,583) at 37°C, 5% CO_2_ in a 24-well culture plate. After 6 h of incubation, the cells were harvested and stained with FITC-conjugated anti-mouse CD4 antibody (BD Pharmingen, #553,046). After the cells were washed, the cells were fixed and permeabilized by BD cytofix/cytoperm plus fixation/permeabilization kit (BD Pharminge, #554,715). The cells were intracellularly stained with PE-Cy5.5-conjugated anti-mouse IFN-γ antibody (BD Pharmingen, # 560,660), PE-conjugated anti-mouse IL-17A antibody (BD Pharmingen, # 560,436) and APC-conjugated anti-mouse IL-4 antibody (BD Pharmingen, # 554,436). For the Tregs, suspended unstimulated CD4^+^ T cells were stained with FITC-conjugated anti-mouse CD4 antibody and APC-conjugated anti-mouse CD25 antibody (BD Pharmingen, #561,048). After washing, fixing, and permeabilizing, the cells were intracellularly stained with PE-conjugated anti-mouse Foxp3 antibody (BD Pharmingen, #560,414). Then, the cells were measured on a FACS-Calibur flow cytometer. The data were analyzed by FlowJo software. For gating, we used isotype staining. The isotype control we used are as followed: PerCP-Cy™5.5 Rat IgG1, κ Isotype Control(BD Pharmingen, # 554,436), PE Rat IgG1, κ Isotype Control (BD Pharmingen, # 554,685) and APC Rat IgG1, κ Isotype Control (BD Pharmingen, # 554,686). For Th1, Th2 and Th17 cells, we selected lymphocytes based on their forward and side scatter properties. Then we gated CD4^+^ IFN-γ^+^ Th1 cells, CD4^+^ IL-4^+^ Th2 cells and CD4^+^ IL-17^+^ Th17 cells based on their fluorescence intensity, respectively. For Treg cells, we selected lymphocytes and CD4^+^ Th cells. Then we gated CD25^+^ Foxp3^+^ Treg cell based on their fluorescence intensity. The experiment was repeated 3 times for each sample.

### Real-time PCR

Total RNA was obtained with TRIzol Reagent (Invitrogen, # 10,296,010) and then converted into cDNA with a RevertAid RT Reverse Transcription Kit (Thermo fisher, # K1691) according to the manufacturer’s instructions. Real time-polymerase chain reaction (RT-PCR) was performed with a LightCycler® 480 System (Roche) using SYBR Green I Master (Roche, # 04707516001). The primer sequences are as follows: CVB3: F-GTCTGCCTGCGTTTATTTC, R-ACTCAGCGTATCGTTTGGA and GAPDH: F-AGGGAAATCGTGCGTGACAT, R-CATCTGCTGGAAGGTGGACA. The relative gene expressions were normalized to the level of GAPDH mRNA transcripts and quantified by the 2-△△CT method. The experiment was repeated 3 times for each sample.

### Western blot

The total proteins of heart and spleen were lysed in Radio Immunoprecipitation Assay (RIPA) Lysis Buffer (Beyotime Biotechnology, #P0013B), which contained PMSF (Beyotime Biotechnology, #ST506). A bicinchoninic (BCA) protein assay kit (Thermo fisher, #23,225) was used to detect the protein concentration. Samples containing 40 μg of protein were separated on a 10 to 15% SDS-PAGE gel and transferred to a polyvinylidene (PVDF) membrane. The membranes were blocked for 2 h with 5% nonfat milk powder at room temperature. Then, the membranes were incubated with primary antibody at 4℃ overnight, and they were incubated with a secondary HRP-conjugated antibody (1:5000, biosharp, #BL002A) for 2 h on the second day. Then, the membranes were detected with ECL. The protein bands were scanned, and the band density was calculated using AlphaEaseFC software. GAPDH was used as a control to calculate the expression of all proteins. The antibodies used included the following: mouse anti-T-bet monoclonal antibody (1:1000, Abcam, #ab91109), rabbit anti-GATA3 polyclonal antibody (1:1000; Abcam, #ab106625), rabbit anti-RORγ polyclonal antibody (1:1000; Abcam, # ab207082), goat anti-IL-1 monoclonal antibody (1:1000; Cell Signaling Technology, #50,794), goat anti-IL-6 monoclonal antibody (1:1000; Cell Signaling Technology, #12,912), rabbit anti-TNF polyclonal antibody (1:1000; Abcam, #ab6671), rat anti-IL-4 polyclonal antibody (1:1000; Abcam, #ab11524), rabbit anti-IL-17 polyclonal antibody (1:1000; Abcam, # ab79056), rabbit anti-Foxp3 polyclonal antibody (1:1000; Abcam, # ab54501) and rabbit anti-GAPDH polyclonal antibody (1:1000; Cell Signaling Technology, # 5174). The experiment was repeated 3 times for each sample.

## Statistics

Data are expressed as the mean±S.D. The Kaplan-Meier method was used to analyze the survival rate. Comparisons of each group were performed by one-way analysis of variance (ANOVA) and the Least – Significant Difference (LSD). The differences in the pathological scores were evaluated using a Mann–Whitney U-test. Analysis was performed with SPSS 17.0 statistical software for Windows. A value of p < 0.05 was considered significant.
